# Descriptive and molecular epidemiology of leishmaniasis diagnosed from clinical samples in the United States, 2021-2022

**DOI:** 10.1128/spectrum.01055-24

**Published:** 2024-09-09

**Authors:** Thao T. Truong, Karissa Crawford, Ichih Wang-McGuire, Kendal Jensen, Aisha Mushtaq, Nicole A. P. Lieberman, Frederick S. Buckner, Wesley C. Van Voorhis, Brad T. Cookson, Stephen J. Salipante, Joshua A. Lieberman

**Affiliations:** 1 Department of Laboratory Medicine and Pathology, University of Washington School of Medicine, Seattle, Washington, USA; 2 Center for Emerging and Re-emerging Infectious Diseases (CERID), Division of Allergy & Infectious Diseases, Department of Medicine, University of Washington School of Medicine, Seattle, Washington, USA; 3 Department of Microbiology, University of Washington School of Medicine, Seattle, Washington, USA; Memorial Sloan Kettering Cancer Center, New York, New York, USA

**Keywords:** epidemiology, *Leishmania*, PCR, molecular epidemiology, diagnostics, parasitology

## Abstract

Leishmaniasis is a rare disease in the United States, with an estimated annual incidence of dozens of cases occurring primarily in travelers, migrants, and military personnel. True disease incidence is unknown, since leishmaniasis is not a nationally notifiable condition. Here, we describe the results of molecular leishmaniasis over a 1-year interval (September 2021 to August 2022) when our laboratory served as the primary national reference laboratory for molecular diagnosis of civilian leishmaniasis. We tested 218 specimens submitted from 36 states yielding 94 of the 186 (50.5%) positive cases with species or species complex-level identification and 18 novel mini-exon alleles. Most species belonged to subgenus *Viannia* (75.6%) and associated with cutaneous or mucocutaneous disease. Cases were associated with recent travel (18.1%), travel timing unspecified (7.4%), migration (7.4%), remote travel (2.1%), military (1.1%), or unknown history (63.8%). These data illustrate the clinical utility of molecular testing for leishmaniasis and provide unique insight into disease epidemiology.

**IMPORTANCE:**

Leishmaniasis is a disfiguring, neglected parasitic infection endemic to the Southern United States and the Americas. Despite significant populations at risk—travelers, military and foreign service members, and migrating persons—the epidemiology of the disease in the United States is poorly understood. Moreover, few clinical laboratories in the United States can test for the disease. Here, we present results from 1 year of testing for this disease at a major reference laboratory. These findings are particularly relevant because they coincide with a temporary “pause” on all clinical testing at the CDC. Our findings suggest at least several hundred cases occur each year in the United States. In particular, mucosal leishmaniasis may be more common than previously reported. We also highlight greater genetic diversity in *Leishmania* species endemic to the Americas than has been previously sampled, with implications for diagnostic specificity.

## INTRODUCTION

*Leishmania* parasites are transmitted to humans by bites from infected phlebotomine sandflies. Over 20 *Leishmania* species are described and cause a wide spectrum of human diseases. Although leishmaniasis is considered a neglected tropical disease, it is endemic to 98 countries and its worldwide distribution is impacted by human conflict, travel, immigration, and displacement (1). *Leishmania* species endemic to the Eastern hemisphere have been geographically categorized as “Old World” *Leishmania* (OWL) whereas those endemic to the Western hemisphere have been termed “New World” *Leishmania* (NWL) (2). Most human infection is caused by species from one of two subgenera. The New World subgenus *Viannia* includes *L. braziliensis* spp. complex and *L. guyanensis* spp. complex, which cause cutaneous and/or mucocutaneous lesions. Subgenus *Leishmania* includes Old World *L. major*, Old World *L. tropica* spp. complex, and New World *L. mexicana* spp. complex, which all cause cutaneous leishmaniasis (CL). The *Leishmania* subgenus additionally includes *L. donovani* (OWL) and *L. infantum* (OWL)/*L. chagasi* (NWL), which are primarily associated with organotropic visceral leishmaniasis (VL), the most fatal clinical manifestation.

Diagnostic testing methods include histopathology to visualize intracellular amastigotes, *in vitro* culture from lesions, serology, and molecular detection of parasite DNA (3). Molecular methods are considered most sensitive, and are often most rapid (2–5). The University of Washington (UW) reference laboratory developed and validated a polymerase chain reaction (PCR) and Sanger sequencing assay to detect and identify *Leishmania* from tissue and body fluid specimens. The approach interrogates the organism’s multicopy mini-exon gene using primer binding sites conserved across *Leishmania* species (6). The amplified region is sufficiently diverse to allow taxonomic determination of species or species complex (6, 7). Along with patient immune status, type and severity of clinical disease, and travel history, knowledge of the infecting *Leishmania* species helps determine the risk for mucocutaneous leishmaniasis (MCL), which impacts treatment and management (3).

*Leishmania* has been considered endemic to the United States since 2015, after reports of autochthonous CL (8–10). Previous studies of *Leishmania* infection epidemiology in the United States have been limited in scope and often lack species-level identification, comprising surveillance of armed forces members, case reports, and multicenter observational studies (11–13). From September 2021 to July 2022, culture, serology, and molecular testing and identification of *Leishmania* species were temporarily discontinued at the U.S. Centers for Disease Control and Prevention (CDC) as part of an agency-wide pause on clinical testing (14), leaving the UW diagnostic reference laboratory the primary service providing molecular *Leishmania* testing to the general US population during this period (3). This situation offers a unique and relatively unbiased opportunity to examine molecular testing results for patients with suspected leishmaniasis within the United States. Here, we present the largest survey of *Leishmania* testing in the United States performed to date, encompassing 94 positive patient cases with species-level identification from testing specimens from 186 patients submitted over a 1-year period. Our work lends significant insights into the descriptive and molecular epidemiology of leishmaniasis in the United States.

## MATERIALS AND METHODS

### Assay validation

We validated a PCR and Sanger sequencing assay targeting the *Leishmania* mini-exon gene (6) under the CLIA ’88 regulatory framework and guidelines established by the Clinical Laboratory Standards Institute. Assay performance was assessed using seven cultured isolates (axenic promastigote culture) and genomic DNA purchased from the American Type Culture Collection (ATCC 50129); 13 residual clinical specimens incidentally positive for *Leishmania* spp. by 28S sequencing from broad-range fungal PCR performed in our laboratory and/or by culture and molecular analysis at the CDC Parasitic Disease Branch; and 22 residual clinical specimens negative for *Leishmania* spp. but positive for other pathogens (Table S1). DNA extraction, sequence analysis, and case review were performed as previously described (15, 16). The assay uses previously described primers targeting the mini-exon gene and uses the following cycling parameters (Applied Biosystems): initial denaturation at 98°C for 2 min, followed by 35 cycles of 97°C for 1 min, 50°C for 0.5 minutes, and 72°C for 1 min, with a final extension of 72°C for 3 min (6). Specimens were tested in technical duplicate with positive, negative, and inhibition control reactions. Assay performance was fully reproducible across three operators.

### Patient population

The study cohort included all patients with leishmania testing performed at the UW Molecular Microbiology clinical diagnostic laboratory between the assay’s initial offering on 1 Septembr 2021 and 31 August 2022. The CDC referred leishmaniasis testing to the University of Washington from October 2021 to July 2022. Acceptable specimen types included fresh and formalin-fixed paraffin-embedded (FFPE) tissue, body fluids, buffy coat preparations, and (with laboratory director approval) stain-positive peripheral blood.

### Demographic and clinical information

Demographic data, including sex, age, state from which specimen was submitted, dates of collection/submission, and specimen description/anatomic site, were obtained from the laboratory information system. When available, clinical history, histopathology findings, and travel history were obtained from medical records including physician clinicopathologic consultations.

### Data analysis

Data were analyzed in R (version 4.2.1) (17). Maps and figures were generated using usmap, tidyverse, cowplot, and ggtree packages (18–22). “Cases” were defined as unique patients, regardless of the number of specimens tested. Chi-square tests were used to compare categorical variables using GraphPad QuickCalcs (23).

Assembled mini-exon sequences were MAFFT aligned in Unipro Ugene v44.0 (24) with published reference sequences representing human-infecting species complexes and curated manually to consolidate mini-exon alleles exhibiting minor length variation. Trees were generated by IQ-Tree with automatic substitution model optimization (25, 26) and visualized with ggtree (27).

## RESULTS

### Assay validation

We determined assay performance for a PCR and Sanger sequencing assay targeting the *Leishmania* mini-exon gene (6) using extracted DNA from axenic cultures and residual clinical specimens known to be positive for *Leishmania* spp. or common and/or morphologically similar pathogens (Table S1). The assay’s 95% limit of detection (LOD) was established as one genome per reaction by testing serial dilutions of purified genomic DNA into testing matrix (Table S2). Sensitivity of testing for DNA extracted from cultured promastigote cell pellets, purified parasite DNA, and patient specimens was 90.5%. Species complex identification was fully concordant with CDC gold-standard testing. Specificity was 100%, with no cross-reactivity with human DNA or other tested pathogens (Table S1).

### Clinical cohort

**Fig 1:**
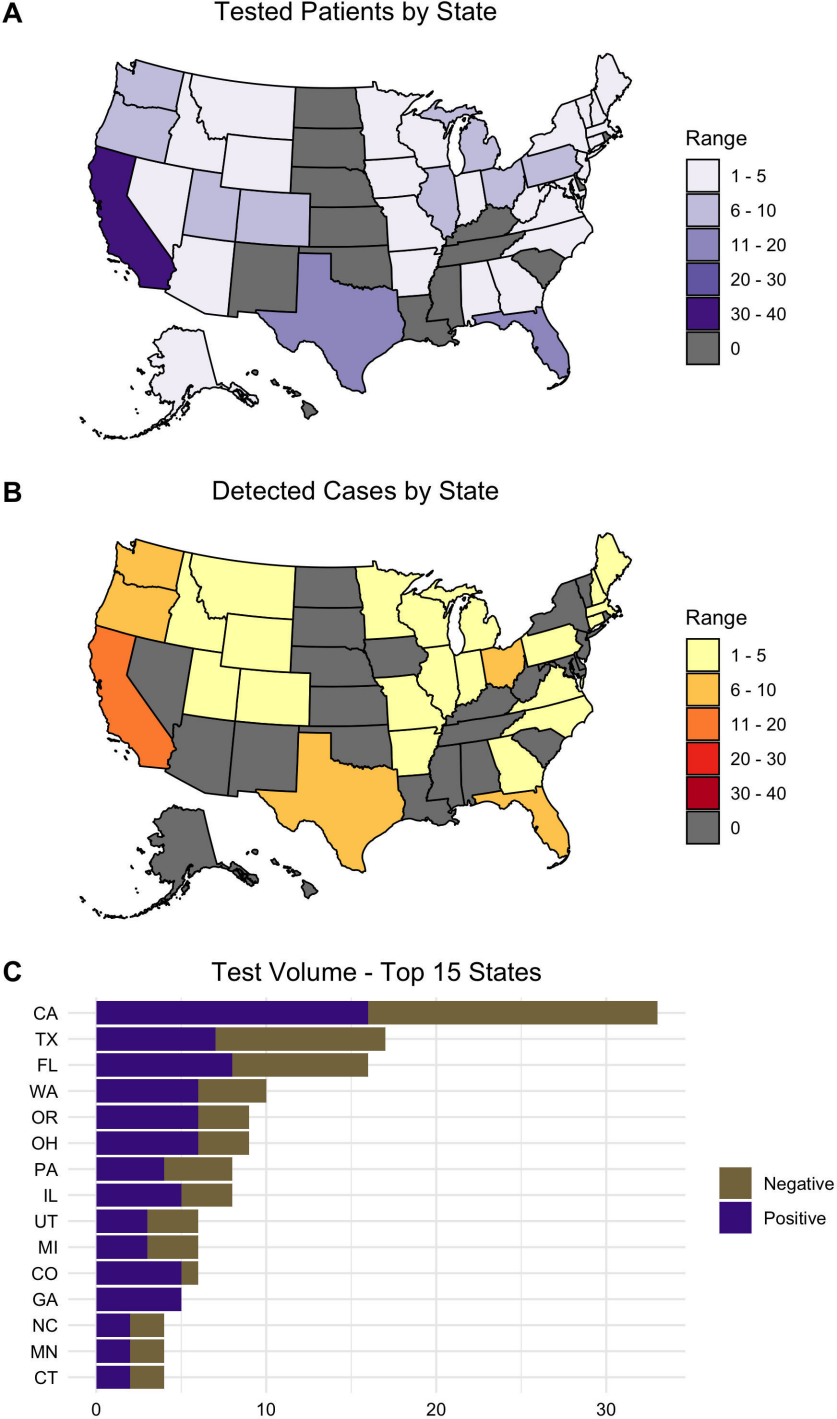
Geographic distribution of patients whose specimens were submitted to our laboratory for *Leishmania* testing from September 2021 to August 2022. Not depicted in this figure: one case from Quebec, Canada, one case from Puerto Rico. One case from the Dominican Republic was submitted by an institution in Florida. (**A**) The number of patients tested by each state in the United States. (**B**) The number of patients who tested positive for *Leishmania* by each state. (**C**) List of the top 15 states by total test volume.

We tested 218 specimens from 186 patients over the 1-year period from September 2021 through August 2022. Ninety-fourpatients (50.5%) had at least one specimen positive for *Leishmania* (Table 1). Specimens were submitted from 36 US states and Puerto Rico; Quebec, Canada; and the Dominican Republic (Fig. 1). California, Florida, and Texas submitted the most cases for testing, with corresponding positivity rates of 48.5% (16/33), 50.0% (8/16), and 41.2% (7/17) (Fig. 1C). Case and specimen positivity rates were 50.5% and 47.7% over the study period (Tables 1 and 2). The months with the highest case volumes were June, May, and February 2022 (Fig. S1). Median test turnaround time was 3.19 days (IQR 1.32–3.91) from specimen receipt.

**TABLE 1:** Demographic characteristics of patients with positive *Leishmania* PCR results

Characteristic	# Cases tested	# Cases positive	(%) Positive by characteristic	(%) Positive out of total positive cases (*n* = 94)
Total tested	186	94	(50.5)	(100.0)
Age group				
<20	30	19	(63.3)	(20.2)
<10	15	11	(73.3)	(11.7)
20–39	60	34	(56.7)	(36.2)
40–59	57	25	(43.9)	(26.6)
60–79	35	15	(42.9)	(16.0)
>80	4	1	(25.0)	(1.1)
Sex				
Male	107	60	(56.1)	(63.8)
Female	76	32	(42.1)	(34.0)
Unknown	3	2	(66.7)	(2.1)
Travel history				
Travel history				
Migration	10	7	(70.0)	(7.4)
Travel ≤6 months	21	17	(81.0)	(18.1)
Travel >6 months	4	2	(50.0)	(2.1)
Travel, timing not specified	13	7	(53.8)	(7.4)
Military	1	1	(100.0)	(1.1)
No travel history	1	0	(0)	(0)
Unknown	136	60	(44.1)	(63.8)

**TABLE 2:** Clinical and histopathological correlation with test positivity

	Positive for *Leishmania* (%)
Specimen type (*n* = 218)	
Fresh tissue	39/111 (35.1)
FFPE Total	65/107 (60.7) 104/218 (47.7)
Cases with ulcers or lesions on physical exam (*n* = 186)	
Yes	45/68 (66.1)
Unknown	49/118 (41.5)
Cases with histopathological findings	
Amastigotes reported	15/17 (88.2)
Staining suspicious for organisms No organisms seen	12/12 (100.0) 27/61 (34.4)
Unknown	40/96 (41.7)

### Demographics of positive cases

Of patients testing positive for *Leishmania*, 59 of the 94 (62.8%) were aged 20–59 and 19 (20.2%) were under the age of 18, including 11 under age 10 (Table 1; Fig. S2). Sixteen patients (17.0%) were over age 60, including 1 patient over 80 (Table 1; Fig. S2). More patients with leishmaniasis were male (63.8%) (Table 1). Travel history was unavailable for 60 positive cases (63.8%), while 17 (18.1%) had documented international travel to *Leishmania*-endemic regions within 6 months prior to testing. Two (2.1%) cases documented international travel ≥5 years prior to diagnosis,and 7 (7.4%) noted travel history without specifying timing. Twelve positive cases (12.8%) were associated with travel to Costa Rica (Table S3). Seven cases (7.4%) had a history of migration from endemic areas and one case (1.1%) from a military service member with *L. major*. Although we did not receive cases where the possibility of endemic infection was explicitly documented, we noted two *L. mexicana* spp. complex infections from Texas, where that species is endemic, although travel information was not provided.

### Specimen characteristics

Despite expectations for DNA degradation, formalin-fixed paraffin-embedded (FFPE) specimens yielded a higher overall positivity rate than fresh tissue specimens: 60.7% of 107 specimens versus 35.1% of 111 specimens (*P* < 0.0002, Chi-square test; Table 2) (28). Positivity was highest (100%) in cases with staining patterns suspicious for intracellular organisms (Table 2). Cases where characteristic *Leishmania* amastigotes were noted on histopathology had the second-highest positivity rate (88.2%) (Table 2). The two histopathology-positive, molecular-negative cases, were instead positive for either *Staphylococcus aureus* or *Histoplasma capsulatum* by additional clinical molecular testing performed in our laboratory.

**Fig 2:**
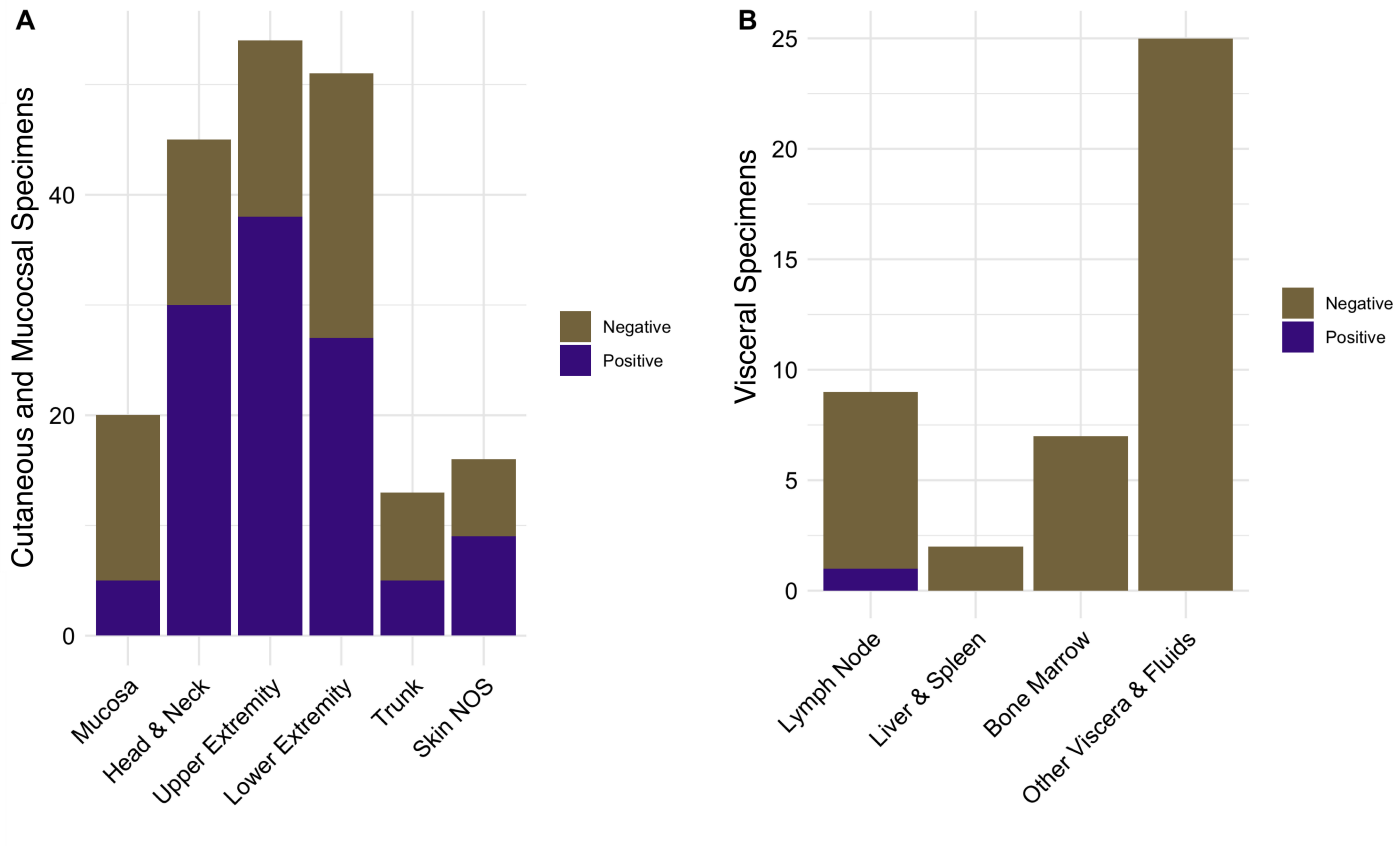
Specimen types submitted for *Leishmania* testing. Number of negative and positive specimens submitted from (**A**) specimens from skin and mucosal sites; and, (**B**) specimens from viscera and body fluids. Anatomic sites for “Other viscera & fluids” included pulmonary (three lung, two bronchoalveolar lavage), uncategorized bone & soft tissue (4), ascitic fluid (1), colon (1), CNS (seven brain, three CSF), and ocular (one eye, one vitreous fluid), subacromial space (1), and retroperitoneal ganglion (1). NOS = Not otherwise specified.

The most commonly involved body sites were skin, representing 86 cases (91.5%), particularly from exposed areas (Fig. 2A). Four positive cases (4.3%) were detected from five oropharyngeal specimens and identified as *L. braziliensis* spp. complex, two of which were recurrent infections (Table S4). Only one specimen collected from internal organ sites or body fluids potentially associated with visceral disease tested positive: a paraffin-embedded inguinal lymph node positive for *Leishmania donovani* spp. complex (Fig. 2B).

Twenty-two patients had multiple specimens submitted, comprising 13 positive and 9 negative cases (Table S4). Of the positive cases, nine were specimen pairs collected less than 30 days apart. In three cases, the initial specimen was positive, but subsequent specimens sent >30 days later were negative. One case involved multiple specimens collected 5 months apart that were positive for *L. guyanensis* spp. complex, with the same mini-exon sequence (Table S4). Two instances showed mini-exon sequence variation across different body sites: one case of *L. braziliensis* spp. complex in both an epiglottis and pharynx lesion and one case of *L. guyanensis* spp. complex in both an ear (helix) and shoulder lesion.

### Leishmania species and molecular epidemiology

**Fig 3:**
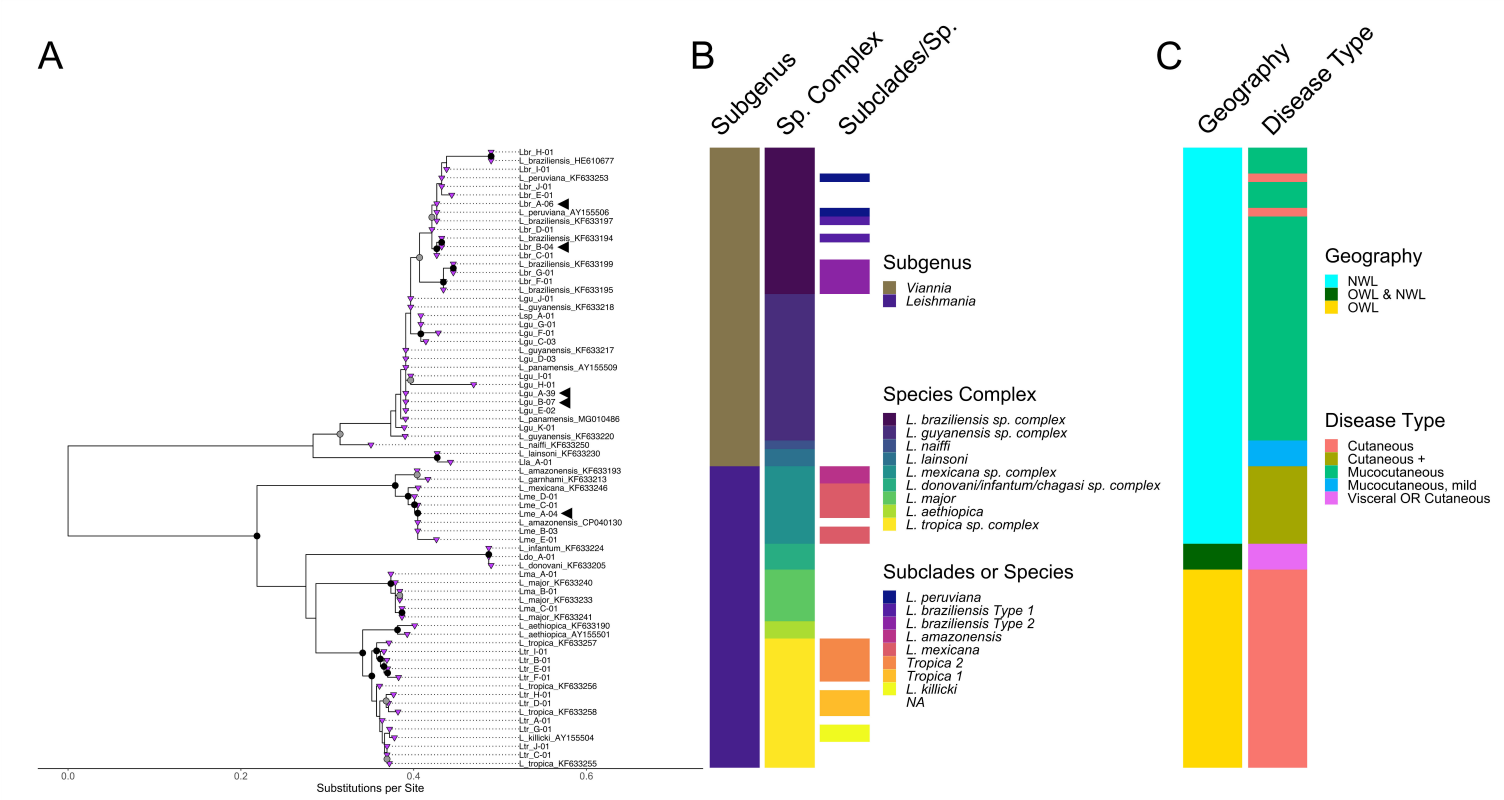
Taxonomic and epidemiologic characteristics of *Leishmania* mini-exon sequences. (**A**) Detected and reference mini-exon sequences are displayed with tips as purple triangles. Sequences from this study have tips labeled with L plus first two letters of species name, sequence variant (upper case letter), and number of times detected. Publicly available sequences tip labels include full species name and mini-exon Genbank accession. The five most abundant mini-exon alleles are marked with arrowheads. Bootstrap values >95% are indicated as solid black nodes and 85–95% as gray nodes with black borders. *L. amazonensis* strain UA301 is indicated by the corresponding mini-exon locus CP040130. *X*-axis scale is substitutions per site. (**B**) Bars represent defined taxonomic descriptions of subgenus and species complex, as well as possible subclades or specific species within each complex as determined by mini-exon sequence similarity. (**C**) Epidemiologic associations, including geography and clinical presentation. Cutaneous + indicates potential for disseminated cutaneous disease. OWL = Old World *Leishmania*, NWL = New World *Leishmania*.

Mini-exon sequencing provided adequate resolution (>80% bootstrap) to achieve species complex-level identification (Fig. 3; Table 3). Of the 94 positive cases, 71 (75.5%) were subgenus *Viannia*. Fifty-three (56.4%) were identified as *L. guyanensis* spp. complex, 17 (18.1%) as *L. braziliensis* spp. complex, and 1 (1.1%) as *L. lainsoni* spp. complex. Detected sequences included 18 previously unreported mini-exon alleles (Table S5, BioProject 974035).

**TABLE 3:** Species identified by mini-exon sequencing

*Leishmania* species (*n* = 94)	# Positive (%)	
Subgenus *Viannia*		
*Leishmania braziliensis* spp. complex	17	(18.1)
*Leishmania guyanensis* spp. complex	53	(56.4)
*Leishmania lainsoni*	1	(1.1)
Subgenus *Leishmania*		
*Leishmania donovani* spp. complex	1	(1.1)
*Leishmania major*	3	(3.2)
*Leishmania mexicana* spp. complex	9	(9.6)
*Leishmania tropica* spp. complex^*a*^	10	(10.6)

^*a*^ One case was reported as *L. killicki*, a member of the *L. tropica* spp. complex.

We observed significant mini-exon sequence conservation within *L. braziliensis*. Two *L braziliensis* spp. complex alleles accounted for 10 of the 17 cases (Lbr_A-06, Lbr_B-04; Fig. 3A, arrowheads) and were separated from each other by three polymorphisms. Sequence conservation across *L. braziliensis* and *L. peruviana* reference strains precluded the resolution of these two species with two exceptions. Two mini-exon sequences, Lbr_F and Lbr_G, formed a clade (>95% bootstrap) with “Type 2” *L. braziliensis* mini-exon reference sequences (KF633199, MHOM/PE/03/LH2511; KF633195, MHOM/CO/90/LEM2216). Travel history was not available for Lbr_F, while Lbr_G had traveled to Peru. These Type 2 *braziliensis* sequences were detected once each.

The two member species of the *L. guyanensis* spp. complex, *L. guyanensis* and *L. panamensis*, had nearly identical previously published mini-exon sequences, precluding identification to the species rank. A single mini-exon allele (Lgu_A-39; Fig. 3A, arrowheads) accounted for most *L. guyanensis* spp. complex cases (33/53), including six cases with identical sequences recovered in multiple lesions. The second most abundant allele (Lgu-B-07; Fig. 3A, arrowheads) accounted for seven cases and was only distinguished from Lgu_A-39 by homopolymer length variation. The next two most abundant alleles (Lgu_C-03, Lgu_D-03) accounted for six additional cases.

Nine cases (9.6%) were *L. mexicana* spp. complex and two alleles accounted for seven of these (Lme_A-04 and Lme_B-03; Fig. 3A, arrowheads). The detected sequences formed a high-confidence clade with *L. mexicana* reference sequences (Fig. 3). However, the mini-exon sequence (CP040130) from one recently completed, unpublished genome of *L. amazonensis*, UA301 (GCA_005317125.1) had 100% identity to multiple detected sequences. The UA301 mini-exon sequence thus defies the otherwise clear separation of *L. mexicana* and *L. amazonensis*/*L. garnhami* into two clades that are well-resolved by mini-exon sequences (Fig. 3).

Fewer cases corresponded to OWL: 10 (10.6%) cases of *L. tropica* spp. complex and 3 (3.2%) *L*. *major*, one of which was from a military service member. Other patients positive for OWL include one patient who traveled to Yemen, one who had previously lived in and recently visited Tunisia, and one identified as an Afghan refugee (Table S3). Available clinical history for the one *L. donovani* spp. complex infection (1.1%) was not sufficient to determine its acquisition and molecular diagnosis could not resolve the organism to the species level. Thus, this case could represent either an infection with Old World species *L. donovani* or *L. infantum*, or infection with New World *L. chagasi*/*infantum*.

Sequences within the *L. tropica* spp. complex included two potential subclades with >80% bootstrap support (Fig. 3A and B). The remaining allele (Ltr_G-01) had 99.75% identity over 392 nucleotides to *L. killicki* (GenBank AY155504) and was derived from a patient who had lived in and recently visited Tunisia. This case was reported to the species rank given the geographic association with *L. killicki* (Fig. 3A; Table S3) (29).

The distribution of identified species paralleled the travel history in the 34 cases with documented exposures. Of these, 23 (67.6%) had traveled to or from North, Central, or South America (Table 3; Table S3). There were seven known migration-related cases, including six NWL infections: five *L. guyanensis* spp. complex and one *L. braziliensis* spp. complex. Four involved patients had traveled from Central and/or South America, while one had immigrated from West Africa, but did not have explicitly documented travel history to an endemic area. One Afghan patient tested positive for *L. tropica* spp. complex (Tables S3 and S5).

## DISCUSSION

Our work describes molecular reference testing for *Leishmania* in the general US population during a period when it was otherwise unavailable. This provides a unique opportunity to compile a comprehensive, 1-year catalog of patient and pathogen characteristics that help approximate the incidence and epidemiology of infections in the United States. In agreement with prior studies involving US travelers, our case demographics reflect a slight male predominance at 63.8% (12, 30–32). Also consistent with previous observations, positive cases were associated with recent travel to Central and South America, particularly Costa Rica (Table 1; Table S2) (12, 30, 31, 33). 11.7% of cases in this work were in children younger than 10 and 6.4% in the 10–18 age range, adding to reports of pediatric infection in the United States (12, 13, 31, 34, 35).

Migration-related leishmaniasis has increased globally, but US-specific data have been limited to sporadic reports (30, 36–39). This work identifies seven cases (7.4% of all positive) of likely migration-related infection originating from multiple continents, highlighting a need for increased clinician awareness in this vulnerable population (Table 1; Table S3). Given that travel histories were not available for most (63.8%) cases, this likely underestimates the true disease burden in this group. Endemic infection with *L. mexicana* spp. complex has been documented, but the provided clinical histories from our cases precluded assessing the probability of autochthonous leishmaniasis.

CL is the most common clinical manifestation reported in recent US studies (11–13), and 91.5% of cases in this study were CL. We additionally detected *L. braziliensis* in four patients with mucosal disease, including two with relapsed MCL acquired during travel several years prior. In contrast, a prior case series and review reported only six documented civilian cases of MCL in the United States from 1975 to 2020 (38). Our cohort may be enriched for MCL due to the high prevalence of subgenus *Viannia*, which accounted for 75% of all cases.

Identification to species complexes within subgenus *Viannia* is clinically informative for treatment and monitoring, due to the associated risk of progression to MCL with most *Viannia* species (3). Within *Viannia*, mini-exon sequencing typically lacked resolution beyond the species complex level, particularly *L. guyanensis* spp. complex (Fig. 3). Consistent with prior analyses (6), our assay could distinguish *L. braziliensis* Type 2 from *L. peruviana* (Fig. 3). Most Type 1 *L*. *braziliensis* and *L. peruviana* could not be distinguished; clinically, this may overestimate a patient’s risk of developing mucosal disease.

Intriguingly, we identified a high-confidence clade of *L. braziliensis* Type 1 (KF633199, Lbr-B, Lbr-C) that may be separable from *L. peruviana* and an outlier sequence in *L. braziliensis* spp. complex (Lbr_H) with 100% identity to the first reported *L. braziliensis* infection in Suriname (HE610677, Fig. 3) (40). These emerging clades suggest additional mini-exon sequences from well-characterized strains could increase molecular diagnostic resolution, particularly in combination with detailed travel history. We note several patients with positive cutaneous specimens who traveled to multiple countries and were consequently at risk for infection from different *Leishmania* species. In such instances improved species-level identification would more accurately assess risk for progression to mucosal involvement (3). However, intermediate forms possibly representing interspecies hybrids between *L. braziliensis* and *L. peruviana* have been reported, and current molecular diagnostic methods may need to be re-evaluated once there is better taxonomic clarification in this area (41).

Visceral leishmaniasis cases are rare in the United States but have been documented in service members returning from endemic regions, and there are concerns for undetected asymptomatic infection and risk for reactivation in these veterans (11, 12, 42). In this series, only one case tested positive for *L. donovani* spp. complex, which is associated with visceral disease and consistent with detection in a lymph node. In agreement with previous studies (6) our assay could not distinguish between *L. donovani* and *L. infantum/chagasi*; however, in the United States the recommended treatment of choice for visceral leishmaniasis regardless of species is liposomal amphotericin B (3).

Clinical and laboratory diagnosis of leishmaniasis can be challenging, and molecular methods are considered the most sensitive modality (2, 3). This work and others have demonstrated the utility of the mini-exon gene as a sensitive, specific, and effective target for distinguishing the major species complexes (6, 7, 43). Other targets, such as ITS1, *hsp70*, 7SL RNA, and kinetoplast minicircle DNA have been evaluated. Comparative studies have shown that all of these targets have different limitations with distinguishing particular closely related species; however, *hsp70* and mini-exon sequences may offer the best resolution (6, 44). Targeting the mini-exon gene is advantageous in a clinical laboratory setting due to the high copy number (100s/genome) compared to *hsp70* (1–15/genome), thus maximizing sensitivity (45). The amplicon is also smaller and more practical to amplify and sequence compared to *hsp70*, which enables better throughput and recovery, particularly from fragmented DNA in FFPE specimens (6).

Our assay readily detects infection from a variety of specimen types associated with CL, MCL, and VL, and has demonstrated good performance with FFPE specimens, which are a convenient option to test in scenarios where fresh tissue is not available without re-biopsy of the patient. Although fresh tissue is generally favored over FFPE due to concerns that formalin can damage DNA, we observed a higher positivity rate in FFPE specimens (28). This may reflect a higher pre-test probability with specimens that have undergone prior histologic review. This is consistent with our high positivity rate in specimens submitted with a pathology report noting potential organisms seen on stain. With rapid turnaround times (~3 days), molecular testing and sequencing can effectively inform patient treatment and management.

We found that mini-exon sequencing is sufficient for the identification of clinically relevant species complexes, supporting prior work showing that species identification from mini-exon analysis had comparable performance to multilocus sequence typing or single gene target assays (6, 43). We successfully resolved *L. lainsoni* from other *Viannia* species, which has historically posed technical challenges (6). However, an important limitation is that the most closely related species within each of the *L. donovani*, *L. guyanensis*, and *L. braziliensis* species complexes could not be resolved. This challenge is clinically relevant within *L. braziliensis* spp. complex, given the greater propensity of *L. braziliensis* for mucocutaneous spread relative to *L. peruviana* (46). Nevertheless, expanding publicly available mini-exon sequence records may enable more specific identification.

We identified 18 previously unreported mini-exon alleles, expanding the diversity of known sequences and highlighting opportunities to improve molecular species identification. Whole-genome sequencing (WGS) will enable more accurate and specific identification, resolve technical artifacts, and identify co-infections (7). The challenges in resolving species within complexes and unexpected findings from recently sequenced strains, such as *L. amazonensis* UA301 clading with *L. mexicana* strains, emphasize the evolutionary complexity of *Leishmania* species (41, 47, 48).

Although illuminating, there are limitations to this study. First, our estimates of disease burden and the granularity of our demographic data are limited by the incompleteness of clinical information provided to our reference laboratory. We do not require this information upon specimen submission due to the logistical burden this would impose on outside institutions. The limited clinical data also poses challenges in interpreting patients with multiple positive specimens, as prolonged PCR positivity does not necessarily reflect treatment failure (49). In the cases where travel history was provided, we cannot definitively confirm the respective infections were acquired in the reported locations, although all were consistent with the identified species. Second, it is likely that additional civilian cases were not tested at our laboratory, particularly after testing resumed at CDC in July 2022. Third, *Leishmania* (50); therefore, the single *L. major* case we detected in a US armed forces personnel may underrepresent disease burden. A survey by Stahlman et al. documented over 2,000 cases in the US Armed Forces from 2001 to 2016 but noted significantly fewer cases starting in 2011, with only (3, 11, 50). Finally, mini-exon sequence data have inherent limitations. The *Leishmania* mini-exon gene is comprised of 100–200 tandem repeats, which increases the risk of chimeric PCR artifacts (7) and masks low abundance sequence variants. Although our validation studies confirmed this issue did not interfere with the ability to discriminate between different species complexes, we are limited in making more refined phylogenetic inferences that would require more comprehensive WGS.

Leishmaniasis remains challenging to recognize clinically, and we expect it is still underdiagnosed in the United States (2, 12, 13). Most state public health agencies do not require positive test notification, with the exception of Texas, and suboptimal reporting compliance has been documented (13). Our study demonstrates that molecular testing by mini-exon sequencing provides clinically actionable species-level identification, and when considered in aggregate, suggests a significantly higher annual incidence in the United States than previously appreciated. The burden, distribution, and biology of this disease will continue to change with global travel, migration, and climate changes impacting parasite, vector, and host geography (51). This work highlights the role of molecular testing in enabling diagnostic laboratory professionals, providers, and public health agencies to track and appropriately treat this disease.

## Data Availability

Sequences have been deposited to Genbank (accessions OR026045-OR026086) with metadata available in BioSample accessions SAMN35159702 - SAMN35159803 (BioProject PRJNA974035). Deidentified, patient-specific molecular data are available upon request with appropriate human subject approvals.

## SUPPLEMENTAL MATERIAL

Supplemental material spectrum.01055-24-s0001.docx Fig. S1 and S2; Tables S1 to S5.

ASM does not own the copyrights to Supplemental Material that may be linked to, or accessed through, an article. The authors have granted ASM a non-exclusive, world-wide license to publish the Supplemental Material files. Please contact the corresponding author directly for reuse.
