# Supplementary material for: Descriptive and molecular epidemiology of leishmaniasis diagnosed from clinical samples in the United States, 2021-2022
Source: Microbiol Spectr. 2024 Sep 9;12(10):e01055-24. doi: 10.1128/spectrum.01055-24 (PMC11448060; doi:10.1128/spectrum.01055-24)
Supplement: Supplemental material — Fig. S1 and S2; Tables S1 to S5. [file spectrum.01055-24-s0001.docx]

**Supplemental Materials**

**Figure S1. Case volume and positivity by month of testing.**


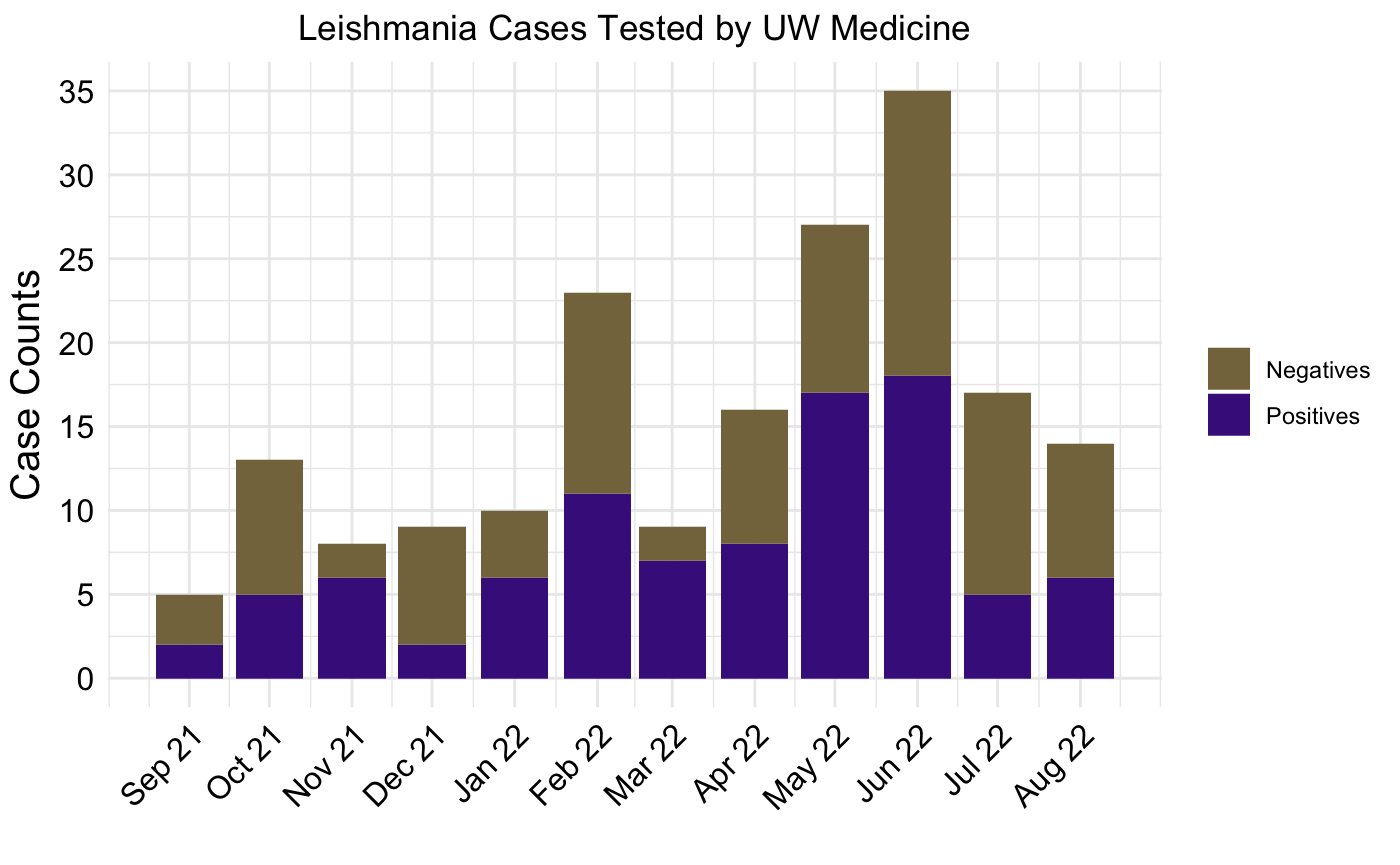
 **Figure S1.** Number of negative and positive specimens submitted and tested during each month of the study period. Negative cases are in gold; positive cases in purple.

**Figure S2. Age Distribution of Patients Tested for Leishmaniasis.**

**
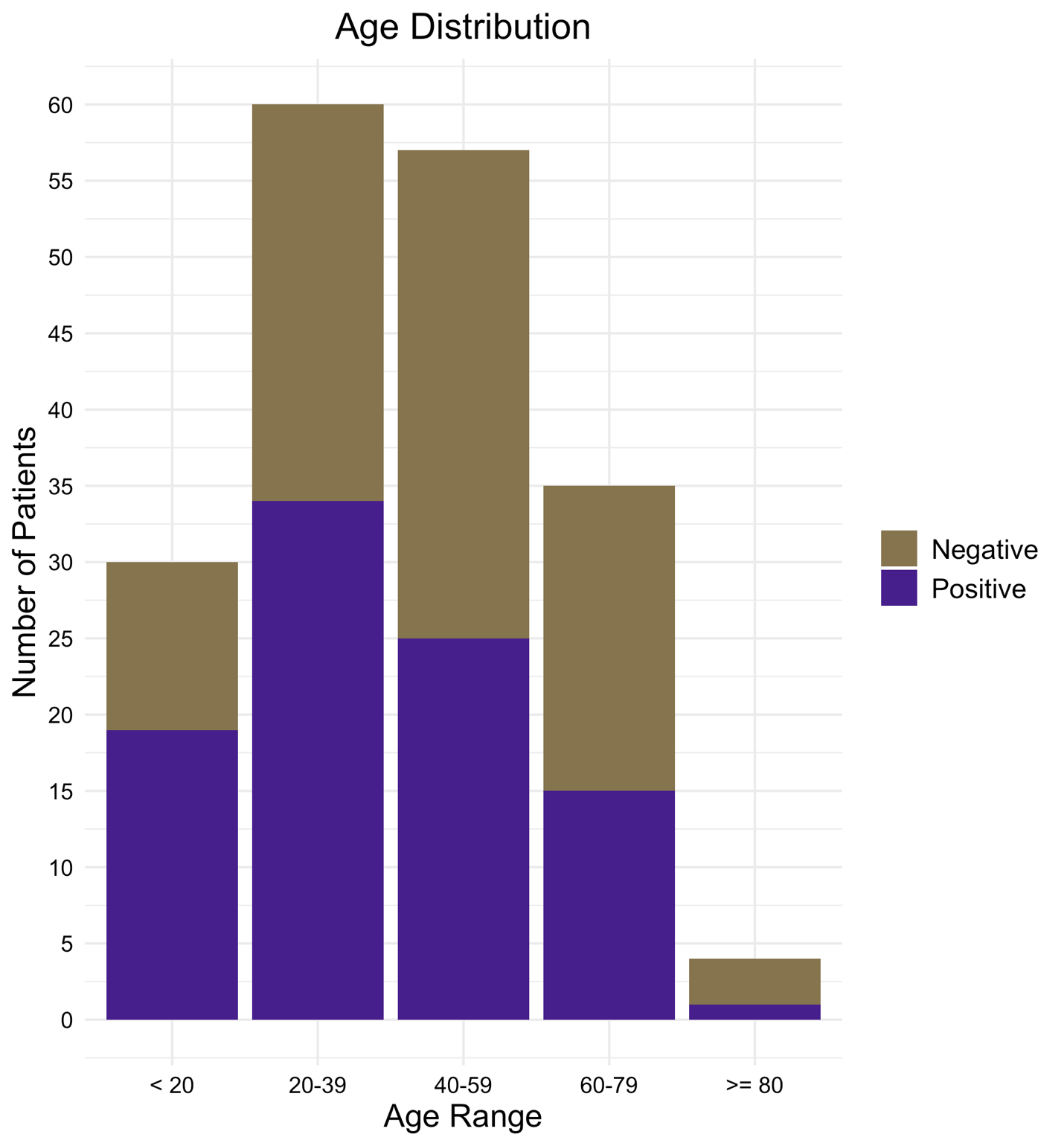
**

**Figure S2.** Number of patients tested by indicated age range (X-axis). Negative cases are in gold; positive cases in purple.

**Table S1. Test performance and validation**

| Specimen number | Specimen type | Reference identification | *Leishmania* PCR identification | Agreement |
| --- | --- | --- | --- | --- |
| 1 | Cultured strain | *L. donovani* | *L. donovani* spp. complex | Yes |
| 2 | Cultured strain | *L. major* | *L. major* | Yes |
| 3 | Cultured strain | *L. aethiopica* | *L. aethiopica* | Yes |
| 4 | Cultured strain | *L. chagasi* | *L. donovani* spp. complex | Yes |
| 5 | Cultured strain | *L. guyanensis* | *L. guyanensis* | Yes |
| 6 | Genomic DNA | *L. tropica* | *L. tropica* | Yes |
| 7 | Cultured strain | *L. panamensis* | *L. guyanensis/L. panemensis* | Yes |
| 8 | Cultured strain | *L. amazonensis* | *L. amazonensis* | Yes |
| 9 | Patient specimen FFPE | *L. tropica* | *L. tropica* | Yes |
| 10 | Patient specimen FFPE | *L. braziliensis* | *L. braziliensis* | Yes |
| 11 | Patient specimen FFPE | *L. braziliensis* | *L. braziliensis* | Yes |
| 12 | Patient specimen FFPE | *L. braziliensis* | *L. braziliensis* | Yes |
| 13 | Patient specimen FFPE | *L. braziliensis* | Not detected | No |
| 14 | Patient specimen FFPE | *L. braziliensis* | *L. braziliensis* | Yes |
| 15 | Patient specimen FFPE | *L. braziliensis* | *L. braziliensis* | Yes |
| 16 | Patient specimen FFPE | *L. braziliensis* | Not detected | No |
| 17 | Patient specimen FFPE | *Leishmania sp.* | *L. chagasi/L. infantum* | Yes |
| 18 | Patient specimen tissue | *Leishmania sp.* | *L. guyanensis/L. panamensis* | Yes |
| 19 | Patient specimen tissue | *Leishmania sp.* | *L. guyanensis/L. panamensis* | Yes |
| 20 | Patient specimen FFPE | *Leishmania sp.* | *L. guyanensis/L. panamensis* | Yes |
| 21 | Patient specimen tissue | *Leishmania sp.* | *L. braziliensis/L. peruviana* | Yes |
| 22 | Patient specimen fluid | *Pneumocystis jiroveci* | Not detected | Yes |
| 23 | Patient specimen fluid | *Pneumocystis jiroveci* | Not detected | Yes |
| 24 | Patient specimen tissue | *Toxoplasma gondii* | Not detected | Yes |
| 25 | Patient specimen tissue | *Toxoplasma gondii* | Not detected | Yes |
| 26 | Patient specimen tissue | *Histoplasma capsulatum* | Not detected | Yes |
| 27 | Patient specimen tissue | *Histoplasma capsulatum* | Not detected | Yes |
| 28 | Patient specimen FFPE | *Histoplasma capsulatum* | Not detected | Yes |
| 29 | Patient specimen fluid | *Histoplasma capsulatum* | Not detected | Yes |
| 30 | Patient specimen fluid | *Candida albicans* | Not detected | Yes |
| 31 | Patient specimen FFPE | *Candida tropicalis* | Not detected | Yes |
| 32 | Patient specimen fluid | *Candida glabrata* | Not detected | Yes |
| 33 | Patient specimen tissue | *Sporothrix schenckii* | Not detected | Yes |
| 34 | Patient specimen tissue | *Aspergillus fumigatus* | Not detected | Yes |
| 35 | Patient specimen FFPE | *Aspergillus fumigatus* | Not detected | Yes |
| 36 | Patient specimen tissue | *Escherichia coli* | Not detected | Yes |
| 37 | Patient specimen FFPE | *Escherichia coli* | Not detected | Yes |
| 38 | Patient specimen tissue | *Pseudomonas aeruginosa* | Not detected | Yes |
| 39 | Patient specimen tissue | *Pseudomonas aeruginosa* | Not detected | Yes |
| 40 | Patient specimen fluid | *Staphylococcus aureus* | Not detected | Yes |
| 41 | Patient specimen fluid | *Staphylococcus aureus* | Not detected | Yes |
| 42 | Patient specimen tissue | *Streptococcus pneumoniae* | Not detected | Yes |
| 43 | Patient specimen fluid | *Streptococcus pneumoniae* | Not detected | Yes |

**Table S2. Limit of detection**

| Species | Replicates positive at 1 genome/reaction |
| --- | --- |
| *L. major* | 4/4 |
| *L. donovani* | 2/2 |
| *L. aethiopica* | 6/6 |
| *L. chagasi* | 6/6 |
| *L. tropica* | 4/4 |
| *L. panamensis* | 6/6 |
| *L. amazonensis* | 4/4 |
| **Total** | **32/32 (100%)** |

**Table S3. Species identified by travel history**

| **Country** | **Travel history < 6 mo.** | **Travel history > 6 mo.** | **Travel history, timing not specified** | **Migration** | **Military** | **Species detected** |
| --- | --- | --- | --- | --- | --- | --- |
| Central America | | | | | | |
| Belize | 1 |  |  |  |  | *L. mexicana* spp. complex (1) |
| Costa Rica | 9 |  | 3 |  |  | *L. guyanensis* spp. complex (11) *L. braziliensis* spp. complex (1) |
| Guatemala |  |  |  | 1 |  | *L. braziliensis* spp. complex (1) |
| Panama | 1 |  |  |  |  | *L. guyanensis* spp. complex (1) |
| Not otherwise specified |  |  | 1 |  |  | *L. guyanensis* spp. complex (1) |
| North America | | | | | | |
| Mexico |  |  | 1 |  |  | *L. mexicana* (1) |
| South America | | | | | | |
| Bolivia |  | 1^A^ |  |  |  | *L. braziliensis* spp. complex (1) |
| Ecuador | 2 |  |  | 1 |  | *L. guyanensis* spp. complex (2) *L. lainsoni* (1) |
| Peru | 1 |  |  |  |  | *L. braziliensis* spp. complex (1) |
| Guyana |  | 1 |  |  |  | *L. braziliensis* spp. complex (1) |
| Venezuela |  |  |  | 1 |  | *L. guyanensis* spp. complex (1) |
| Middle East | | | | | | |
| Afghanistan |  |  |  | 1 |  | *L. tropica* spp. complex (1) |
| Yemen | 1 |  |  |  |  | *L. tropica* spp. complex (1) |
| Africa | | | | | | |
| Nigeria |  |  |  | 1 |  | *L. guyanensis* spp. complex (1) |
| Tunisia | 1^B^ |  |  |  |  | *L. killicki* (1) |
| Other | | | | | | |
| Travel noted, location unknown |  |  | 1 |  | 1 | *L. major* (1) *L. braziliensis* spp. complex (1) |
| Multiple endemic countries | 1 |  | 1 | 2 |  | *L. guyanensis* spp. complex (4) |

^A^ Patient had documented infection after traveling in 2016 and had relapse of mucocutaneous leishmaniasis in 2022.

^B^ Patient lived in Tunisia prior to coming to the U.S. and developed infection after a recent return visit.

**Table S4. Repeat testing**

| Category | # patients |
| --- | --- |
| I. Multiple specimens negative |  |
| a. 30+ days apart | 3 |
| b. Within 30 days | 6 |
| II. Multiple specimens positive within 30 days |  |
| a. Different sites | 4 |
| b. Similar or same site | 4 |
| c. Unknown if sites are same or different | 1 |
| III. Initial positive specimen, subsequent negative specimen 30+ days later | 3 |
| IV. Multiple specimens positive 30+ days apart | 1 |

Table S5: Metadata for Positive Cases

| **Study Number^1^** | **Age** | **Sex** | **Species Identified** | **Location Acquired** | **Anatomic Site** | **Mini-Exon Sequence** | **Allele Comments** |
| --- | --- | --- | --- | --- | --- | --- | --- |
| 200jB | 30-34 | M | L. braziliensis spp. complex | Not provided | Skin, Arm | Lbr_B-04 |  |
| 3NY5V | 40-44 | M | L. braziliensis spp. complex | Not provided | Skin, Foot | Lbr_E-01 | Previously unreported |
| 3pZcY | 15-19 | M | L. guyanensis spp. complex | Not provided | Skin, Leg | Lgu_F-01 | Previously unreported |
| 4AGIK | 15-19 | M | L. mexicana spp. complex | Not provided | Skin, Face | Lme_A-04 |  |
| 4qXFx | 60-64 | M | L. guyanensis spp. complex | Not provided | Skin, Arm | Lgu_A-39 |  |
| 4qXFx | 60-64 | M | L. guyanensis spp. complex | Not provided | Skin, Arm | Lgu_A-39 |  |
| 4Tpmt | 65-69 | M | L. guyanensis spp. complex | Not provided | Skin, Leg | Lgu_A-39 |  |
| 5d8uv | 50-54 | M | L. guyanensis spp. complex | Not provided | Skin, Arm | Lgu_A-39 |  |
| 5N4y7 | 25-29 | M | L. guyanensis spp. complex | Not provided | Skin, Back | Lgu_E-02 |  |
| 6DvUz | 25-29 | M | L. guyanensis spp. complex | Not provided | Skin, Hand | Lgu_D-03 |  |
| 71Qur | 30-34 | M | L. guyanensis spp. complex | Not provided | Skin, Leg | Lgu_B-07 |  |
| 7lE4E | 25-29 | M | L. guyanensis spp. complex | Costa Rica | Skin, Leg | Lgu_A-39 |  |
| 7nBtA | 30-34 | M | L. guyanensis spp. complex | Not provided | Skin, Face | Lgu_C-03 | Previously unreported |
| 9s3Zi | 20-24 | M | L. mexicana spp. complex | Not provided | Skin, Leg | Lme_B-03 |  |
| A1UVR | 50-54 | F | L. guyanensis spp. complex | Costa Rica | Skin, Hand | Lgu_A-39 |  |
| aFZBo | 50-54 | M | L. guyanensis spp. complex | Nigeria | Skin, Leg | Lgu_B-07 |  |
| ahPNz | 15-19 | M | L. guyanensis spp. complex | Multiple endemic countries: Costa Rica OR Belize | Skin, Chest | Lgu_A-39 |  |
| ahPNz | 15-19 | M | L. guyanensis spp. complex | Multiple endemic countries: Costa Rica OR Belize | Skin, Elbow | Lgu_A-39 |  |
| aNewR | 5-9 | F | L. major | Not provided | Skin, NOS | Lma_B-01 |  |
| ASuG7 | 55-59 | M | L. guyanensis spp. complex | Costa Rica | Skin, Leg | Lgu_A-39 |  |
| Bb1ZK | 50-54 | F | L. mexicana spp. complex | Not provided | Skin, Leg | Lme_B-03 |  |
| bJ2RQ | 65-69 | M | L. guyanensis spp. complex | Not provided | Skin, NOS | Lgu_C-03 | Previously unreported |
| bVQ6L | 5-9 | M | L. tropica spp. complex | Afghanistan | Skin, Face | Ltr_A-01 | Previously unreported |
| bz3MQ | 25-29 | M | L. guyanensis spp. complex | Costa Rica | Skin, NOS | Lgu_A-39 |  |
| cvuHr | 40-44 | U | L. lainsoni | Ecuador | Skin, Arm | Lla_A-01 | Previously unreported |
| dDNho | < 5 | M | L. tropica spp. complex | Not provided | Skin, Face | Ltr_H-01 | Previously unreported |
| dl8Uh | < 5 | F | L. guyanensis spp. complex | Panama | Skin, Leg | Lgu_A-39 |  |
| dOJ9R | 35-39 | M | L. braziliensis | Not provided | Skin, Knee | Lbr_F-01 | Type 2 L. braziliensis |
| DqThk | < 5 | F | L. mexicana spp. complex | Not provided | Skin, Face | Lme_D-01 | Previously unreported |
| dxbWJ | 20-24 | M | L. guyanensis spp. complex | Not provided | Skin, Arm | Lgu_A-39 |  |
| gdTX9 | 25-29 | M | L. tropica spp. complex | Not provided | Skin, Hand | Ltr_B-01 | Previously unreported |
| gSFeb | 45-49 | M | L. guyanensis spp. complex | Costa Rica | Skin, NOS | Lgu_A-39 |  |
| hliza | 40-44 | M | L. guyanensis sppp. complex | Not provided | Skin, NOS | Lgu_D-03 |  |
| HswPn | >80 | M | L. guyanensis spp. complex | Not provided | Skin, Arm | Lgu_A-39 |  |
| ifG7T | 75-79 | M | L. braziliensis spp. complex | Costa Rica | Skin, Leg | Lbr_B-04 |  |
| j5uY1 | 30-34 | M | L. braziliensis spp. complex | Bolivia | Mucosa, Nasal | Lbr_A-06 |  |
| JD1Fi | 35-39 | F | L. guyanensis spp. complex | Costa Rica | Skin, Arm | Lgu_A-39 |  |
| JfPzu | 35-39 | M | L. guyanensis spp. complex | Not provided | Skin, Face | Lgu_A-39 |  |
| JfPzu | 35-39 | M | L. guyanensis spp. complex | Not provided | Skin, Face | Lgu_A-39 |  |
| KaEnn | 25-29 | M | L. braziliensis spp. complex | Not provided | Skin, Nose | Lbr_B-04 |  |
| L7liP | 40-44 | M | L. braziliensis spp. complex | Guatemala | Skin, Nose | Lbr_C-01 | Previously unreported |
| lqj5v | 10-14 | F | L. braziliensis spp. complex | Not provided | Mucosa, Nasal | Lbr_A-06 |  |
| LY4kh | 25-29 | F | L. guyanensis spp. complex | Not provided | Skin, Leg | Lgu_A-39 |  |
| mj7LX | 15-19 | F | L. guyanensis spp. complex | Costa Rica | Skin, Knee | Lgu_A-39 |  |
| MNaNr | 25-29 | M | L. guyanensis spp. complex | Not provided | Skin, Leg | Lgu_A-39 |  |
| MnFJU | 20-24 | F | L. braziliensis spp. complex | Not provided | Skin, Face | Lbr_J-01 |  |
| MQ3Iq | 30-34 | M | L. guyanensis spp. complex | Not provided | Skin, Leg | Lgu_A-39 |  |
| MqS1M | < 5 | F | L. tropica spp. complex | Not provided | Skin, Face | Ltr_J-01 |  |
| N58sC | 30-34 | M | L. braziliensis spp. complex | Not provided | Skin, NOS | Lbr_A-06 |  |
| Ngbt9 | 10-14 | F | L. tropica spp. complex | Not provided | Skin, Face | Ltr_F-01 | Previously unreported |
| nhxid | 60-64 | F | L. braziliensis spp. complex | Not provided | Skin, Face | Lbr_A-06 |  |
| nLIzV | 5-9 | F | L. tropica spp. complex | Yemen | Skin, Nasal | Ltr_D-01 | Previously unreported |
| OkKEs | 45-49 | F | L. guyanensis spp. complex | Costa Rica | Skin, Leg | Lgu_A-39 |  |
| OkKEs | 45-49 | F | L. guyanensis spp. complex | Costa Rica | Skin, Leg | Lgu_A-39 |  |
| oYo7a | 40-44 | F | L. guyanensis spp. complex | Costa Rica | Skin, Face | Lgu_A-39 |  |
| paee2 | 25-29 | U | L. guyanensis spp. complex | Not provided | Skin, NOS | Lgu_B-07 |  |
| pmg22 | 40-44 | M | L. tropica spp. complex | Tunisia | Skin, Arm | Ltr_G-01 |  |
| PRu3I | 35-39 | M | L. guyanensis spp. complex | Costa Rica | Skin, Arm | Lgu_I-01 |  |
| PRu3I | 35-39 | M | L. guyanensis spp. complex | Costa Rica | Skin, Ear | Lgu_H-01 | Previously unreported |
| q6Y0m | 30-34 | F | L. mexicana spp. complex | Belize | Skin, Arm | Lme_A-04 |  |
| qljLb | 70-74 | M | L. guyanensis spp. complex | Not provided | Skin, Buccal | Lgu_A-39 |  |
| QlW8W | 55-59 | F | L. guyanensis spp. complex | Not provided | Skin, Arm | Lgu_A-39 |  |
| QsNa7 | 70-74 | U | L. mexicana spp. complex | Mexico (Yucatan) | Skin, Arm | Lme_A-04 |  |
| QsNa7 | 70-74 | M | L. mexicana spp. complex | Mexico (Yucatan) | Skin, Arm | Lme_A-04 |  |
| r7Bif | 75-79 | M | L. guyanensis spp. complex | Ecuador | Skin, Ear | Lgu_G-01 | Previously unreported |
| r7Bif | 75-79 | M | Leishmania sp, NOS | Ecuador | Skin, Face | Lsp_A-01 | Previously unreported |
| rHGuZ | 35-39 | F | L. guyanensis spp. complex | Not provided | Skin, Arm | Lgu_A-39 |  |
| rUtEq | 10-14 | F | L. guyanensis spp. complex | Venezuela | Skin, Leg | Lgu_B-07 |  |
| RZyXs | 40-44 | M | L. donovani/infantum/chagasi spp. complex | Not provided | Lymph Node, inguinal | Ldo_A-01 |  |
| SHAjS | 25-29 | M | L. tropica spp. complex | Not provided | Skin, NOS | Ltr_C-01 | Previously unreported |
| SrbZI | 35-39 | M | L. guyanensis spp. complex | Not provided | Skin, Face | Lgu_J-01 |  |
| tnbbZ | 35-39 | M | L. guyanensis spp. complex | Multiple endemic countries: Panama OR Honduras OR Nicaragua OR Colombia OR Guatemala | Skin, Hand | Lgu_B-07 |  |
| U19Vi | 10-14 | M | L. guyanensis spp. complex | Not provided | Skin, Hand | Lgu_A-39 |  |
| uf2WQ | 60-64 | F | L. braziliensis sppp. complex | Not provided | Skin, Hand | Lbr_I-01 | Previously unreported |
| UMVeK | 45-49 | M | L. braziliensis spp. complex | Not provided | Mucosa, Tonsil | Lbr_D-01 |  |
| UMVeK | 45-49 | M | L. braziliensis spp. complex | Not provided | Mucosa, Epiglottis | Lbr_H-01 | Reported once, from Suriname |
| UrDnb | 45-49 | F | L. guyanensis spp. complex | Not provided | Skin, Face | Lgu_A-39 |  |
| usKjM | 35-39 | F | L. guyanensis spp. complex | Not provided | Skin, Arm | Lgu_A-39 |  |
| usKjM | 35-39 | F | L. guyanensis spp. complex | Not provided | Skin, Hand | Lgu_A-39 |  |
| viGq0 | 70-74 | M | L. braziliensis spp. complex | Not provided | Skin, Hand | Lbr_B-04 |  |
| VPME3 | 60-64 | M | L. mexicana spp. complex | Not provided | Skin, Face | Lme_C-01 |  |
| vtbyX | 50-54 | M | L. guyanensis spp. complex | Not provided | Skin, Leg | Lgu_A-39 |  |
| VXYJc | 35-39 | M | L. guyanensis spp. complex | Not provided | Skin, NOS | Lgu_A-39 |  |
| VXYJc | 35-39 | M | L. guyanensis spp. complex | Not provided | Skin, Arm | Lgu_A-39 |  |
| W2523 | 45-49 | F | L. guyanensis spp. complex | Not provided | Skin, Arm | Lgu_K-01 | Previously unreported |
| WdF6F | 25-29 | M | L. major | Not provided | Skin, Leg | Lma_A-01 |  |
| wNI9E | 40-44 | F | L. guyanensis spp. complex | Not provided | Skin, Elbow | Lgu_C-03 | Previously unreported |
| WPNzg | 60-64 | M | L. guyanensis spp. complex | Central America | Skin, Back | Lgu_E-02 |  |
| XATsT | 5-9 | F | L. guyanensis spp. complex | Ecuador | Skin, Arm | Lgu_A-39 |  |
| XEnxF | < 5 | M | L. tropica spp. complex | Not provided | Skin, Face | Ltr_I-01 | Previously unreported |
| XkSqR | 50-54 | M | L. guyanensis spp. complex | Multiple endemic countries: Panama OR Mexico | Skin, Knee | Lgu_B-07 |  |
| XpFLs | 65-69 | M | L. mexicana spp. complex | Not provided | Skin, Face | Lme_B-03 |  |
| yfxW2 | 60-64 | M | L. braziliensis spp. complex | Not provided | Skin, Arm | Lbr_A-06 |  |
| YfZoO | < 5 | F | L. guyanensis spp. complex | Not provided | Skin, Knee | Lgu_B-07 |  |
| YHq5X | 45-49 | F | L. tropica spp. complex | Not provided | Skin, Chest | Ltr_E-01 |  |
| yqxw6 | 55-59 | F | L. mexicana spp. complex | Not provided | Skin, Leg | Lme_E-01 | Previously unreported |
| YRcvQ | 25-29 | M | L. guyanensis spp. complex | Not provided | Skin, Leg | Lgu_A-39 |  |
| yv1zu | 5-9 | F | L. guyanensis spp. complex | Multiple endemic countries: Central & South America (including Amazon region) | Skin, Arm | Lgu_D-03 |  |
| zDVRa | 40-44 | M | L. major | Not provided (suspected) | Skin, Arm and Flank | Lma_C-01 |  |
| zNB0C | 20-24 | M | L. braziliensis | Peru | Skin, Foot | Lbr_G-01 | Type 2 L. braziliensis |
| zOa4f | 35-39 | F | L. guyanensis spp. complex | Not provided | Skin, Hand | Lgu_A-39 |  |
| zr4An | 55-59 | M | L. guyanensis spp. complex | Costa Rica | Skin, Leg | Lgu_A-39 |  |
| zSazm | 25-29 | F | L. guyanensis spp. complex | Not provided | Skin, Hand | Lgu_A-39 |  |
| VmHYS | 65-69 | M | L. braziliensis spp. complex | Guyana | Mucosa, Oropharynx | Lbr_A-06 |  |

^1^Study number is a random 6-digit alphanumeric string which provides a unique, anonymous key for each patient.
